# Selenium in serum and neoplastic tissue in breast cancer: correlation with CEA

**DOI:** 10.1038/sj.bjc.6603292

**Published:** 2006-08-01

**Authors:** K Charalabopoulos, A Kotsalos, A Batistatou, A Charalabopoulos, P Vezyraki, D Peschos, V Kalfakakou, A Evangelou

**Affiliations:** 1 Department of Physiology, Clinical Unit, Medical Faculty, University of Ioannina, 13, Solomou str., 452 21 Ioannina, Greece; 2 Department of Pathology, Medical Faculty, University of Ioannina, Ioannina, Greece; 3 Department of Surgery, Peterborough and Stamford Hospitals, Cambridgeshire, UK

**Keywords:** selenium, breast cancer, carcinoembryonic antigen (CEA)

## Abstract

Trace element selenium (Se) is regarded to be a breast cancer preventive factor involved in multiple protective pathways. In all, 80 women with breast cancer who underwent a radical mastectomy were enrolled in the study. Serum Se and carcinoembryonic antigen levels were measured using a fluorometric and IRMA assay, respectively. Se tissue concentration was determined by a tissue extracting fluorometric assay. For statistical analysis purposes *t*-test was used and *P*-values <0.001 were regarded as statistically significant. Serum Se was 42.5±7.5 *μ*g l^−1^ in breast cancer patients and 67.6±5.36 *μ*g l^−1^ in the age-matched control group of healthy individuals. Serum carcinoembryonic antigen in patients was 10±1.7 U ml^−1^ (normal <2.5 U ml^−1^ in nonsmokers/<3.5 U ml^−1^ in smokers). A statistically significant difference was found for both serum Se and CEA between two groups studied (*P*<0.001). Neoplastic tissue Se concentration was 2660±210 mg g^−1^ tissue; its concentration in the adjacent non-neoplastic tissue was 680±110 mg g^−1^ tissue (*P*<0.001). An inverse relationship between Se and CEA serum levels was found in the two groups studied (*r*=−0.794). There was no correlation between serum/tissue Se concentration and stage of the disease. The decrease in serum Se concentration as well as its increased concentration in the neoplastic breast tissue is of great significance. These alterations may reflect part of the defence mechanisms against the carcinogenetic process.

Breast cancer is regarded as the third most common malignancy in humans and the second, after skin cancer, in women (Greenlee *et al*, 2000). Approximately, 150 000 women in the United States of America are affected every year (Wolff and Weston, 1997). Much knowledge has been accumulated on the aetiology and pathogenesis of this malignancy implicating genetic, hormonal and environmental factors (Simopoulos, 2004). The influence of micronutrient status on breast cancer development in humans is, nowadays, under continuous investigation. It has been postulated that a high intake of micronutrients in diet may prevent carcinogenesis (Lianidou *et al*, 1997; Charalabopoulos *et al*, 2003). Trace elements play an important role in a number of biological processes by activating or inhibiting various enzymes (Garg *et al*, 1994). Selenium (Se) is an essential nonmetallic trace element and its plasma and tissue concentration is regulated by incompletely understood homeostatic mechanisms. Se exists in the body in the form of selenocystein, a component of selenoproteins and has important structural and enzymic roles, such as antioxidant activity (Huang *et al*, 1999, Rayman, 2000; Rayman, 2005). Cellular oxidative damage is a general mechanism for cell and tissue injury. Oxidative stress in the target tissue has been suggested to play an important role in carcinogenic process. Thus, Se may act as an antitumoural agent although more studies are needed to investigate the actual role of antioxidants and their possible relationships with trace elements alterations in the pathogenesis of breast cancer (Bock *et al*, 1991; Gerber *et al*, 1996). Furthermore, it has been shown that Se supplementation decreases the COX-2 protein and PGE-2 levels in colorectal cancer cells. In addition, Se exhibits an antiproliferative effect modulating cellular proliferation in G1 phase in both normal and neoplastic cells impairing the expression of c-*fos* and c-*myc* oncogenes (Kuo *et al*, 2002). However, other prospective studies have failed to confirm these findings (Garland *et al*, 1995; Ghadirian *et al*, 2000). A strong relationship between low-serum Se concentration and increased risk of breast cancer has been documented (Bratakos *et al*, 1990a, 1990b; Mannisto *et al*, 2000; Kuo *et al*, 2002; Lopez-Saez *et al*, 2003).

The aim of the present study was to determine the levels serum Se in breast cancer patients and healthy controls and to correlate them with Se levels in neoplastic tissue.

## MATERIALS AND METHODS

In all, 80 women with infiltrative ductal carcinoma (nonspecific type, stages I–IV) who underwent radical mastectomy, 45±10. 6 years old, pre- and postmenopausal, were enrolled in the study. A female aged matched population group (consisting of 250 individuals) was selected for comparison of the laboratory data (Kallistratos *et al*, 1985; Charalabopoulos *et al*, 2006). In patients, serum Se levels were measured, at the time of diagnosis before any kind of treatment was started, by the fluorometric method (Watkinson's) modified by Thorling. This is a specific and very sensitive method among others (eg phasmatometric, nonflame molecular absorption, netronic activation, volumetric method) detecting Se at a quantity of 0.002 *μ*g and is considered the method of choice throughout Europe (Thorling *et al*, 1986). Whole blood (10 *μ*l) was taken and centrifuged. The samples were preserved at –4°C. Neoplastic and healthy tissue samples surrounding the tumour were taken during operation and preserved in liquid N_2_. To determine Se tissue concentration, a modified extracting fluorometric assay, similar to the above described, was used. Serum CEA levels were measured by IRMA assay, using commercial kits. For statistical analysis purposes, *t*-test was used and *P*-values <0.001 were regarded as statistically significant. Table 1 shows laboratory and statistical data of the groups studied.

## RESULTS

Serum Se was 42.5±7.5 *μ*g l^−1^ in breast cancer patients and 67.6±5.36 *μ*g l^−1^ in the age-matched control group of healthy individuals. Neoplastic tissue Se concentration was 2660±210 mg g^−1^ tissue; its concentration in the adjacent non-neoplastic tissue was 680±110 mg g^−1^ tissue. Compared to control group Se concentration in serum was lower in breast cancer patients (*P*<0.001). A statistical significant difference was also found between Se concentration in neoplastic breast tissue compared to normal tissue samples surrounding the neoplastic area; Se levels were almost four-fold higher in neoplastic tissue. Serum CEA levels in breast cancer patients were 10±1.7 U ml^−1^ (normal <2.5 U ml^−1^ in nonsmokers/<3.5 U ml^−1^ in smokers, the mean concentration in the control group was 2.3 U ml^−1^). An inverse relationship between Se and CEA serum levels was found in the two groups studied (*r*=−0.794). No correlation between serum/tissue Se concentration and stage of the disease was found.

## DISCUSSION

Breast cancer is an important contributor to morbidity and mortality in humans. Trace element Se has been regarded as a breast cancer preventive factor (Kallistratos *et al*, 1989; Medina *et al*, 2001). However, in a large study a nonsignificant relationship between Se supplementation and increased breast cancer risk was shown (Clark *et al*, 1996). Se absorption is a complex, poorly understood process. Studies in rats have provided us with some information but little is known about this mechanism in humans. There is a particular Se-containing gene that encodes for Se-containing proteins and its various polymorphisms may lead to decreased element absorption (Esworthy *et al*, 1995). The basal amount of dietary Se is also debated. Dietary intakes show a large geographic variation mainly due to differences in Se bioavailability. In Greece the Se concentration in 315 examined foods was shown to be lower than other European countries and closer to UK. The daily Se intake of Greeks is estimated to approximately 110 *μ*g (Kallistratos *et al*, 1985; Bratakos *et al*, 1987; Bratakos *et al*, 1990b; Charalabopoulos *et al*, 2006).

Human body Se is incorporated into the polypeptide backbone of some proteins and through them it regulates the cellular antioxidant defense system, DNA damage and protein function. Se also controls cell-mediated immunity and B-cell function. The lower serum Se levels in cancer patients can be attributed to either lower Se intake, to sequestration of this element by the tumour cells or both (Di Ilio *et al*, 1985; Bratakos *et al*, 1990a).

To our best knowledge, the present study is the fourth one in the last 40 years (Pub-Med search) providing information regarding the neoplastic tissue Se concentration in breast cancer patients and the first one providing data for serum/tissue Se concentrations simultaneously, in association with serum CEA levels (Garg *et al*, 1994; Sharma *et al*, 1994; Kuo *et al*, 2002). It is notable that Se concentration in the neoplastic tissue was nearly fourfold greater than in the adjacent normal breast tissue. Our findings regarding serum/tissue Se levels in patients suffering from breast cancer are in agreement with other reports in patients with lung, gastric, renal, colorectal and breast cancer, where an increased concentration of Se in the neoplastic tissue was found (Garg *et al*, 1994; Sharma *et al*, 1994; Burguera *et al*, 1995; Kuo *et al*, 2002; Charalabopoulos *et al*, 2006).

It is not clear whether the increased Se concentration in the cancerous tissue is responsible for the decreased serum Se levels found in these patients or if the decreased serum Se levels precede the development of breast cancer. Another consideration is the significance of the increased Se concentration in neoplastic tissue. An attractive hypothesis is that it is part of the defence mechanisms against the neoplastic process. This concept is greatly supported by the already above-mentioned antioxidant action of Se. The usefulness and the significance of our findings should be clarified with further future investigation.

## Figures and Tables

**Table 1:** Se concentration in serum (*μ*g l^−1^) and breast tissue (*μ*g g^−1^ tissue)

	**A**	**B**	**C**	
	**Serum (*μ*g l^−1^) (*x*±s.d.)**	**Neoplastic tissue (*μ*g g^−1^ tissue)**	**Non-neoplastic tissue (*μ*g g^−1^ tissue) (*x*±s.d.)**	*t*-test ^*^P-value *P*<0.001
I				
Breast cancer patients	42.5±7.5	2660±210	680±110	SS between IA and IIA
II				
Healthy individuals	67.6±5.36	NA	NA	SS between IB and IC

Abbreviations: *x*±s.d., mean value±s.d.; NA, nonapplicable; SS, statistically significant; ^*^P-value <0.001 is considered as statistically significant.
